# Everybody's Page

**Published:** 1889-12-28

**Authors:** 


### Everybody's Page.

The late King of Portugal is stated to have had eleven physicians attending him at the time of his death.

Pea soup will be found by the poor a very nutritious form of food; its value in this respect is recognised by sailors.

In India a preparation called *cinchona febrifuge* is manufactured on the Government cinchona plantations, which is a satisfactory substitute for quinine, and costs much less.

The addition of bi-carbonate of soda to milk in order to assist in its preservation has been found to be dangerous, as it creates a lactate of soda, which is often the cause of continued diarrhœa in infants which it is very hard to stop.

Two parts of the pomegranate tree are used medicinally; the root bark makes a decoction used as a gargle for ulcerated mouth, and the rind of the pretty fruit makes a good astringent for a relaxed throat. Here, then, we have use and beauty combined.

It seems that wherever the European goes he must carry with him the fatal vice of drink. It is not pleasant to be told that the state of Zanzibar town after dark is dangerous owing to the number of drinking-shops, and that the great offenders against the peace are drunken Europeans. And we boast of, and thank God for, our civilisation!

Indifference to pain is generally a characteristic of uncivilised peoples such as the natives of Africa, but Mr. F. L. James has discovered a people who are more than indifferent; they seem to love pain for its own sweet self. The Somalis ought to make good surgeons, for people who implore you to divide tissues, and think you wanting in the milk of human kindness if you refuse to cut slices in their tongues, cannot be troubled with nerves.

In most hospitals an old-fashioned habit of scrubbing the ward floors is still maintained. What would our grandmothers say if they knew that in a few hospitals this plan has been abolished and soap and water are no more seen? As a matter of fact, it is found that infection, etc., is less where ward floors are polished and dry rubbed than where a moist, unhealthy atmosphere is maintained by the soap-and-water process.

During the five years from 1882-1887 the mortality in the St. Petersburg hospitals from typhoid fever, intermittent fever, diphtheria, erysipelas, etc., was a trifle less than in the Paris hospitals, whereas scarlatina, chronic bronchitis, and variola claimed more victims at St. Petersburg. In Russia they are fortunate in having practically no scientific past, and so no tradition and prejudice to overcome. They have thus been enabled to avail themselves of all the latest modern improvements in their hospitals, and to profit by the progress which science has made.

Many workmen in this country have complained with no little bitterness of late of their hard lot and small pay, and have shown their discontent in a practical manner by striking. Some have doubtless had justice on their side, and others are repenting at leisure their action taken in misguided haste. Yet the hardest lot of these men is almost comfort in comparison with the surroundings of their Russian brethren, who are ill fed and worse paid. The minimum of a day's work in Russia is eleven or twelve hours, and more often fourteen or fifteen. In Moscow, where living is very expensive, the wage average is from 28 to 40 francs a month, that of women seldom being more than 24 and often as low as eight francs a month. There is much material for the reformer's hand in Russia.

"Nine day fits" is a term given by nurses to infantile tetanus, which often occurs about nine days after birth.

Eczema is often called baker's itch, grocer's itch, etc., according to the trade of the person who suffers from it.

As there is always a certain element of danger in lead pipes, they should not be used for the purpose of carrying drinking water, nor should water be drunk which has been standing in cisterns lined with lead.

A hospital nurse, says Dr. Horand, must not only be kind and devoted, but must also have scientific knowledge, without which she will be of little help to the doctor, but rather a source of embarrassment and danger to the invalid. This is fully recognised over here.

There has been an unpleasant increase of typhoid fever in Paris since May of the present year, and Dr. Olivier has impressed upon the authorities none too soon the absolute necessity of hastening on the execution of the works for giving a proper water supply to the city.

In his charming book, "A Naturalist in North Celebes," Mr. Sydney J. Hickson writes that he can recommend monkey as an *entrée*—with qualifications. It is not bad, "but it is too tasty and strong for a hungry person." The experience of travellers in Norway leads them to damn goat with the same faint praise.

The natives of Celebes obtain many medicines by the infusion and decotion of herbs and fruits; and, seeing that their doctors are able to extract live lizards and nails (and doubtless hammers also) out of a patient's head when he is suffering from headache, it is not surprising that the natives have greater faith in their own drugs than in those of European pharmacopœias. But Mr. Hickson does "not feel disposed to place too much reliance on their efficacy."

A discussion having arisen recently in Melbourne on the numbers of the homeless poor, about which many loose and vague statements had been circulated, the Charity Organisation Society there instructed their secretary to enquire into and report on the matter. With a view to supplementing the returns thus obtained, a search of the streets of the city at night was determined upon. The result of a search made by the police on every beat, and by an independent party of private searchers, was that five men only were found either sleeping in or wandering about the streets.

The French begging impostor, who has lately been the study of M. Maxime du Camp, does not appear to differ from his brethren on this side of the Channel. His methods are practically the same, and his ingenuity is no less. In Paris there exist begging agencies, where the impostor, who has reduced his trade to a science, can, on payment of a small fee, obtain not only the names and addresses of the most pliable persons to approach, but instructions as to the most profitable manner in which to do so. These people are well called the "diplomats of the pavement."

Once more the doctors disagree. In St. Petersburg it seems that many of the leading men in the profession take a somewhat alarming view of the outbreak of influenza as being the probable forerunner of cholera, which they fear may before long spread into Russia from Mesopotamia. In Moscow the profession does not take such a pessimist view, and the principal doctors there do not agree with the views of their brethren in St. Petersburg, alleging that the connection between outbreaks of influenza and cholera has been mere coincidence. Time will decide.

